# Feasibility of discharge within 72 hours of major colorectal surgery: lessons learned after 5 years of institutional experience with the ERAS protocol

**DOI:** 10.1093/bjsopen/zrac002

**Published:** 2022-02-16

**Authors:** Alberto Biondi, Maria Cristina Mele, Annamaria Agnes, Laura Lorenzon, Marco Cintoni, Emanuele Rinninella, Giuseppe Marincola, Domenico D’Ugo, Antonio Gasbarrini, Roberto Persiani

**Affiliations:** 1 Università Cattolica del Sacro Cuore, Rome, Italy; 2 Dipartimento di Scienze Mediche e Chirurgiche, Fondazione Policlinico Universitario Agostino Gemelli IRCCS, Rome, Italy; 3 Scuola di Specializzazione in Scienza dell’Alimentazione, Università di Roma Tor Vergata, Rome, Italy

## Abstract

**Background:**

Early postoperative discharge after colorectal surgery within the enhanced recovery after surgery (ERAS) guidelines has been demonstrated to be safe, although its applicability has not been universal. The primary aim of this study was to identify the predictors of early discharge and readiness for discharge in a study population.

**Methods:**

Early discharge was defined as discharge occurring in 72 h or less after surgery. The characteristics and clinical outcomes of the patients in the early and non-early discharge groups were compared, and variables associated with early discharge were identified. Additionally, independent variables associated with the readiness for discharge within 48 h were evaluated.

**Results:**

Of 965 patients who underwent colorectal surgery between January 2015 and July 2020, 788 were included in this study. No differences in readmission, reoperation, or 30-day mortality were observed between the early and non-early discharge groups. Both early discharge and readiness for discharge had a positive association with adherence to 80 per cent or more of the ERAS items and a negative association with the female sex, duration of surgery, drain positioning, and postoperative complications.

**Conclusion:**

Early discharge after colorectal surgery is safe and feasible, and is not associated with a high risk of readmission or reoperation. Discharge at 48 h can be reliably predicted in a subset of patients. Future studies should collect prospective data on early discharge related to safety, as well as patients’ expectations, possible organizational issues, and effective costs reduction in Italian clinical practice.

## Teaser

In this study, we investigated the predictors of early successful recovery and discharge after colorectal surgery as a primary aim, and the surgeons’ bias preventing timely discharge as a secondary aim. Our results confirmed that, within the ERAS discharge criteria, a subgroup of patients could be safely discharged within 72 h or less after surgery, that another subgroup was eligible for discharge within 48 h of surgery, and that surgeons’ bias was linked to a perceived high risk for comorbidities, to the complexity of surgery, and to possible management issues, even though these factors had minimal impact on the clinical outcomes.

## Introduction

Colorectal surgery in high-volume centres is associated with low morbidity (17 to 21.3 per cent) and mortality rates (1.2 to 2.2 per cent)^1,2^. The perioperative management of colorectal surgery patients is often optimized by the application of ‘enhanced recovery after surgery’ (ERAS) principles^3^, which have been indicated to reduce the incidence of perioperative complications, the length of hospital stay, and overall costs^4^.

Avoiding an unnecessary hospital stay is one of the goals of the ERAS protocol, which includes readiness-for-discharge criteria to assist clinical decision-making regarding the discharge time of patients. The discharge criteria after colorectal surgery are usually the tolerance of oral nutrition, pain management, bowel recovery, and autonomy in daily living activities^5^. Several studies conducted in high-volume international institutions have also developed the concept of perioperative management optimization, focusing on the safety and feasibility of early discharge (less than 48 to 72 h) after surgery^6–8^ based on these clinical objective discharge criteria. Their aim was to identify populations that can be managed in an outpatient setting, and reducing costs and the burden of prolonged hospital stay. The results have suggested that an early-discharge approach is safe and feasible in selected patients.

Some studies have suggested that in up to 50 per cent of patients, discharge is postponed, even when all discharge criteria are met^9,10^. This finding suggests that the patient discharge time is often influenced by factors not strictly related to postoperative recovery: the healthcare system; surgeon preferences and habits; patient expectations; urbanicity and cohabitation status; insurance; and costs. These factors must be considered and mitigated against to promote an early-discharge policy in different contexts.

This study was designed to describe the current clinical practice regarding the discharge of patients who have undergone colorectal surgery and received perioperative care in accordance with the ERAS protocol. The primary aim was to identify the predictors of early successful recovery and discharge. The secondary aim was to investigate the discrepancy between the actual day of discharge and the day on which patients were considered eligible for discharge according to objective criteria.

## Methods

### Patients

Data from all consecutive patients who underwent elective colorectal surgery for cancer or benign diseases at the General Surgery Unit of the Fondazione Policlinico Universitario A. Gemelli IRCCS (University Teaching and Research Hospital) in Rome, Italy, between January 2015 and July 2020 were retrospectively reviewed. Patients who underwent colorectal surgery, including right and left hemicolectomy, transverse colonic resection, total colectomy, and rectal resection, were included for analysis. Both open and minimally invasive procedures were included. Patients who underwent emergency resection; colorectal resection without anastomosis (i.e. Hartmann or Miles procedures); local transanal excision or transanal minimally invasive surgery; non-resectional procedures such as ileostomy/Hartmann reversal; or isolated stoma creation without resection were excluded from analysis.

### ERAS protocol

All patients admitted to our unit since 2015 and scheduled for elective colorectal resection were treated in compliance with the ERAS protocol, as previously reported^11,12^. Accordingly, all patients underwent preoperative counselling and a full nutritional assessment (bioelectrical impedance analysis, and routine laboratory tests, including complete blood count with formula and creatinine, albumin, cholesterol, transferrin, vitamin B12, and vitamin D levels). Mechanical bowel preparation was only administered to patients undergoing mid and low rectal resection. Preoperative carbohydrate drinks were administered preoperatively. All patients received intravenous antibiotic prophylaxis, antithrombotic prophylaxis with fractionated heparin, and/or elastic stockings according to the Caprini score^13^. All patients underwent active intraoperative warming. Postoperative pain management included fixed-dose intravenous (i.v.)/oral acetaminophen (1 g, three times daily) and i.v./oral ketorolac as required. No postoperative opioid drugs (morphine or tramadol) were administered. A visual analogue scale (VAS) was administered up to the day of discharge. Compliance with ERAS and the number of items fulfilled by each patient were prospectively recorded in the institutional database. The following criteria were used to determine the readiness for discharge (RfD): tolerance to solid food; autonomous mobilization for more than 6 h or return to baseline conditions prior to surgery; adequate pain control (VAS < 4) with oral medication; bowel recovery (time to first flatus or stool) and no evidence of postoperative complications.

### Data collection

Data were extracted from a prospectively created database (Institutional Review Board approval number for the ERAS project: protocol 50958/17 (4876/18) ID: 1808); parameters included age (measured in years), sex, Charlson Comorbidity Index^14^, ASA score, residence (outside the city of Rome, outside the Lazio region), the year of surgery, the type of surgery (right-sided resection, left-sided resection, left hemicolectomy and sigmoidectomy with intraperitoneal anastomosis, transverse resection, rectal resection, total colectomy, other surgeries), postoperative monitoring (including ICU admission), diverting stoma creation, drain positioning, compliance with more than 80 per cent of the ERAS items, postoperative stay (measured in days), postoperative morbidity and mortality, readmission, and reoperation. The postoperative complications were recorded according to the Clavien-Dindo classification^15^, and defined as minor (Clavien-Dindo grade I–II) and major complications (Clavien-Dindo grade III–IV). Postoperative mortality, readmission, and reoperations were defined as events occurring within 30 days of the surgical procedure.

### Definitions and outcomes

Early discharge was defined as discharge occurring in 72 h or less after surgery. The primary outcome was the difference between the characteristics and clinical outcomes of the patients in the early and non-early discharge groups, and the independent variables associated with early discharge (72 h or less postoperatively) were identified. Additionally, the feasibility of an earlier discharge (48 h or less after surgery) was evaluated selecting the independent variables associated with the RfD within 48 h. For the secondary analysis, patients discharged from hospital as soon as they fulfilled the criteria of RfD were defined as ‘dischargeable and discharged’ (DD), while patients not discharged as soon as they fulfilled the criteria were defined as ‘dischargeable not discharged’ (DND).

### Statistical analysis

The clinicopathological characteristics were summarized using frequencies and percentages for the categorical variables and means and standard deviations or medians and ranges for the continuous variables. Patient characteristics were compared using Pearson’s χ^2^ test or Fisher’s exact test for categorical variables and Student’s *t* test for the continuous variables. Multivariable analyses for variables possibly associated with the primary and secondary outcomes were conducted with backward logistic regression, with *P* < 0.1 set as the limit for inclusion in the progressive steps of the regression model. Statistical analysis was conducted using SPSS Statistics for Macintosh, version 25.0 (IBM, Armonk, New York, USA). All statistical tests were two-sided at a significance level of 0.05.

## Results

Between January 2015 and July 2020, 965 patients underwent colorectal surgery in our unit. According to the inclusion and exclusion criteria, 788 patients were selected for inclusion in this study. No patients were lost to follow-up at 30 days. Readiness for discharge was not assessed in 13 of 788 patients. The median length of postoperative stay was 4 days (range 2 to 68 days).

### Early *versus* non-early discharge

The clinicopathological characteristics and short-term outcomes of the patients according to whether they were discharged early (146 patients (18.5 per cent)) or not (642 patients (81.5 per cent)) are described in *Tables 1* and *2*. A progressive increase over time in the rate of patients discharged early (within 72 h) was observed, reaching 28.9 per cent in 2019. There was a significant difference in the rate of early discharge (within 72 h) in patients who underwent different surgical procedures (*P* < 0.001), as 26.5 per cent of patients who underwent right hemicolectomy were discharged within 72 h, followed by 19.5 per cent of patients who underwent left-sided resections, 14.3 per cent of patients who underwent a transverse resection, 9.6 per cent of patients who underwent rectal resection, and 0 per cent of patients who underwent total colectomy. No differences in readmission, reoperation, or mortality at 30 days were observed between the early and non-early discharge groups. Five patients of 788 (0.6 per cent) died postoperatively: mortality occurred in two patients in the more than 72 h discharge group, and three patients died during hospitalization (these three patients were excluded from subsequent analyses). Multivariable logistic regression (*Table 3*) identified a positive association of early discharge (within 72 h) with adherence to 80 per cent or more of the ERAS items and negative associations with female sex, duration of surgery, length of postoperative ICU stay, drain positioning, and postoperative complications.

**Table 1:** Characteristics of patients in the early and non-early discharge groups

Variable	Early discharge (≤ 72 h), *n* = 146 (18.5%)	Non-early discharge > 72 h, *n* = 642 (81.5%)	*P*
**Mean (s.d.) age (years)**	66 (12)	68 (12)	0.089
**Sex**			
Male	85 (20)	339 (80)	0.236
Female	61 (16.8)	303 (83.2)	
**Resident outside Rome**			
No	52 (18.1)	236 (81.9)	0.796
Yes	94 (18.8)	406 (81.2)	
**Resident outside Lazio region**			
No	123 (19.7)	501 (80.3)	0.095
Yes	23 (14)	141 (86)	
**Mean (s.d.) BMI (kg/m^2^)**	25.9 (4.25)	26 (4.26)	0.843
**ASA classification**			
ASA 1–2	121 (18.8)	521 (81.2)	0.540
ASA ≥ 3	21 (16.5)	106 (83.5)	
NA	4 (21)	15 (78.9)	
**Mean (s.d.) CCI**	3.12 (1.66)	3.35 (2.06)	0.209
**Surgical procedure**			
Right colectomy	74 (26.5)	205 (73.5)	0.001
Left colectomy	47 (19.5)	194 (80.5)	
Transverse colectomy	2 (14.3)	12 (85.7)	
Rectal resection	21 (9.6)	198 (90.4)	
Total colectomy	0 (0)	18 (100)	
Other colic resection	2 (11.8)	15 (88.2)	
**Colorectal disease**			
Benign	14 (13.7)	88 (86.3)	0.181
Malignant	132 (19.2)	554 (80.8)	
**Surgical approach**			
Open	6 (5.5)	102 (94.5)	<0.001
Minimally invasive	140 (20.6)	540 (79.4)	
**Year of procedure**			
2015	0 (0)	115 (100)	<0.001
2016	11 (7.4)	137 (92.6)	
2017	32 (21.9)	114 (78.1)	
2018	44 (26.3)	123 (73.7)	
2019	44 (28.9)	108 (71.1)	
2020	15 (25)	145 (75)	
**Mean (s.d.) duration surgery (min)**	170 (56)	224 (74)	<0.001
**Diverting stoma**			
No	139 (21.4)	510 (78.6)	<0.001
Yes	7 (5)	132 (95)	
**Postoperative monitoring in the ICU**			
No	140 (19.8)	568 (80.2)	0.007
Yes	6 (7.5)	74 (92.5)	
**Drain positioning**			
No	109 (35.6)	197 (64.4)	<0.001
Yes	37 (7.7)	445 (92.3)	
**Adherence to ERAS items**			
< 80 (%)	11 (4.7)	222 (95.3)	<0.001
≥ 80 (%)	135 (24.3)	420 (75.7)	

Data are *n* (%) unless otherwise indicated. Percentages are expressed by line. NA, not assessed; CCI, Charlson comorbidity index; ERAS, enhanced recovery after surgery.

**Table 2:** Short-term outcomes of patients in the early and non-early discharge groups

Variable	Early discharge (≤ 72 h), *n* = 146 (18.5%)	Non-early discharge > 72 h, *n* = 642 (81.5%)	*P*
DND*			
No	66 (45.2)	219 (34.3)	0.013
Yes	80 (54.8)	420 (65.7)	
**Postoperative complications**			
No	140 (95.9)	461 (71.8)	<0.001
Yes	6 (4.1)	181 (28.2)	
**Postoperative major complications**			
No	145 (99.3)	615 (95.8)	0.038
Yes	1 (0.7)	27 (4.2)	
**Readmission†**			
No	142 (97.3)	612 (96.1)	0.494
Yes	4 (2.7)	25 (3.9)	
**Reintervention**			
No	145 (99.3)	626 (97.5)	0.175
Yes	1 (0.7)	16 (2.5)	
**Mortality**			
No	146 (100)	637 (99.2)	0.285
Yes	0 (0)	5 (0.8)	

Data are *n* (%) unless otherwise indicated. Percentages are expressed by column. *Three patients died before discharge were excluded from the evaluation. †Five patients in the non-early discharge group were not able to recall if they were readmitted within 30 days from discharge.

**Table 3:** **Multivariable logistic regression for factors associated with early discharge (**≤** 72 h)**

Variable	Beta	*P*	OR	95% c.i.
**Resident outside Lazio**	−0.578	0.057	0.561	0.310 to 1.017
**Female sex**	−0.624	0.005	0.536	0.346 to 0.829
**Duration of surgery**	−0.008	0.000	0.992	0.989 to 0.996
**Postoperative ICU stay**	−0.964	0.049	0.381	0.146 to 0.995
**Drain positioning**	−1.479	0.000	0.228	0.141 to 0.369
**Adherence to ≥ 80% of ERAS items**	1.151	0.003	3.161	1.495 to 6.684
**Postoperative complications**	−2.262	0.000	0.104	0.041 to 0.267

Variables included residence outside Rome, residence outside Lazio, age, sex, BMI, Charlson comorbidity index (CCI), minimally invasive surgery, type of surgery, stoma creation, duration of surgery, postoperative ICU stay, drain positioning, adherence to ≥ 80% of enhanced recovery after surgery (ERAS) items, postoperative complications, and major complications. OR, odds ratio; CI, confidence interval.

### Readiness for discharge within 48 h

According to the RfD criteria, 115 patients (14.8 per cent) were fit for discharge within 48 h. These patients had a lower overall rate of complications than patients who were not fit for discharge within 48 h (4.3 *versus* 26.1 per cent; *P* < 0.001). There were no significant differences in major complication, readmission, reoperation, or mortality rates (*Table 4*). Multivariable logistic regression (*Table 5*) revealed significant positive associations of RfD within 48 h with the presence of a diverting stoma and adherence to 80 per cent or more of the ERAS items, and significant negative associations with female sex, duration of surgery, drain positioning, and postoperative complications.

**Table 4:** Short-term outcomes of patients who were ready for discharge (RfD) at 48 h or not

Variable	RfD at 48 h, *n* = 115 (14.8%)	Not RfD at 48 h, *n* = 660 (85.2%)	*P*
**Postoperative complications**			
No	110 (95.6)	488 (73.9)	<0.001
Yes	5 (4.3)	172 (26.1)	
**Major postoperative complications**			
No	114 (99.1)	639 (96.8)	0.230
Yes	1 (0.9)	21 (3.2)	
Readmission*			
No	111 (96.5)	633 (96.2)	1
Yes	4 (3.5)	25 (3.8)	
**Reintervention**			
No	114 (99.1)	646 (97.9)	0.711
Yes	1 (0.9)	14 (2.1)	
**Mortality after discharge†**			
No	115 (100)	658 (99.7)	1
Yes	0 (0)	2 (0.3)	

Data are *n* (%). Percentages are expressed by column. *Three patients who died before discharge were excluded from the evaluation. †Two patients in the group not RfD at 48 h were not able to recall if they were readmitted within 30 days from discharge.

**Table 5:** Multivariable logistic regression for factors associated with readiness for discharge at 48 h

Variable	Beta	*P*	OR	95% c.i.
**Female sex**	−0.717	0.002	0.488	0.311 to 0.765
**Minimally invasive surgery**	1.086	0.086	2.964	0.857 to 10.253
**Diverting stoma**	0.911	0.026	2.486	1.114 to 5.546
**Duration**	−0.007	0.001	0.993	0.988 to 0.997
**Drain positioning**	−1.006	0.000	0.366	0.221 to 0.605
**Adherence to ≥** **80% of ERAS items**	1.141	0.008	3.131	1.344 to 7.290
**Postoperative complications**	−2.119	0.000	0.120	0.043 to 0.338

Variables included residence outside Rome, residence outside Lazio, age, sex, BMI, Charlson comorbidity index (CCI), minimally invasive surgery, type of surgery, stoma creation, duration of surgery, postoperative ICU stay, drain positioning, adherence to ≥** **80% of enhanced recovery after surgery (ERAS) items, postoperative complications, major complications. OR, odds ratio.

### ‘Dischargeable not discharged’ *versus* ‘dischargeable and discharged’

The DND rate was 54.8 per cent in the 72 h early-discharge group and 65.7 per cent in the non-early-discharge group (*Table 2*). There were no significant differences between DD and DND patients with regard to readmission, reoperation, or mortality after discharge (*Table S1*).

Multivariable logistic regression conducted in patients with no evidence of postoperative complications (*Table S2*) showed a significant association of right hemicolectomy and a tendency towards significance for rectal resection (*P* = 0.065) with DD status, and significant associations of minimally invasive surgery and Charlson comorbidity index (CCI) with the DND status.

## Discussion

This study was carried out in a large cohort of patients who underwent colorectal surgery with the ERAS protocol, identifying a subgroup of patients that were safely discharged within 72 h or less after surgery, and a subgroup of patients potentially eligible for discharge within 48 h postoperatively. Discharge delay in patients ready for discharge was also investigated, and some of the possible reasons for discharge delay were identified.

The primary analysis identified factors associated with early discharge (within 72 h): male sex; a short duration of surgery; no postoperative ICU stay; no drain placement; and strong adherence to the ERAS items. Similarly, factors predictive of RfD within 48 h were male sex, minimally invasive surgery, the presence of a diverting stoma, short duration of surgery, no drain placement, and a strong adherence to the ERAS items. Both early discharge (within 72 h) and RfD within 48 h showed a satisfactory safety profile, with few postoperative complications, and risk levels for major complications, readmission, reoperation, and mortality that were not significantly higher compared with patients not discharged early.

The safety and feasibility of reducing the length of in-hospital stay after colorectal surgery is a matter of intense debate. Initial studies have reported a worrisome increased rate of readmission in patients discharged within 48 h, accounting for up to 27 per cent and 20 per cent in patients undergoing open and laparoscopic surgery, respectively^16^, while a lower readmission rate (11.33 per cent *versus* 20.1 per cent) was reported in patients with a LOS of 3 days *versus* 2 days^17^. Nevertheless, recent studies reported a low rate of readmission in patients discharged within 48 h or even at 24 h, especially after a laparoscopic approach with the use of enhanced-recovery protocols^6,17,18^. A recent Italian single-centre RCT assessed the safety and feasibility of discharging patients on postoperative day 2 after laparoscopic colectomy and after the first flatus passage, in the absence of complication-related symptoms^19^. In another recent paper summarizing the experience of two high-volume colorectal centres in the USA and Switzerland, 13.4 per cent of patients were discharged within 48 h. This approach was associated with patient age of less than 60 years, a lower ASA grade, restrictive fluid management, a shorter duration of surgery, and a minimally invasive approach. The early-discharge group was associated with lower rates of postoperative complications, major complications, and reintervention^8^.

In our study, similar predictive factors for early discharge within 72 h and early RfD were identified, such as surgery duration and the application of the ERAS items. However, there were differences, as we found female sex to be a negative predictive factor for both early discharge (72 h) and early RfD (48 h). Drain positioning (related to the complexity of surgery, and possible impact on patient’s mobilization) had a negative impact on both early discharge (72 h) and early RfD. In the secondary analysis, among the whole cohort of patients, concordance between the day of actual discharge and the day of RfD was detected for only approximately one-third of patients. This result suggests that length of stay may be influenced by several non-clinical factors that invariably postpone hospitalization. Length of stay and RfD are two well-known measures of recovery after surgery that have been validated in colorectal surgery^18^, and are often used to determine the success of the ERAS protocol. In this study, RfD was recorded as a variable in our prospectively maintained database on the ERAS protocol, providing a more objective definition of successful recovery after surgery. The results of our study also revealed a higher rate of concordance between the day of actual discharge and the day of RfD in patients undergoing right hemicolectomy and rectal resection, and a higher rate of discrepancy in patients with a high CCI, those undergoing minimally invasive surgery, and those with a diverting stoma. The reasons why these characteristics are associated with delayed discharge are not fully understood. Some potential explanations could be that patients undergoing minimally invasive surgery may have a shorter recovery than after open surgery. Patients with comorbidities may have been managed with a higher level of caution. Finally, the presence of a diverting stoma is usually associated with more complex surgeries with higher-risk anastomoses, and in-hospital time is required to provide appropriate education on stoma management postoperatively. Conversely, patients undergoing right colectomy and patients undergoing rectal resection were usually discharged in a timely manner. This finding probably reflects the surgeon’s confidence with the specific surgical technique and the low perceived risk associated with ileocolic anastomosis and rectal resections without a diverting ileostomy^20,21^.

The main implication of this study is that some of the reasons for which patients are not discharged as soon as they are fit for discharge should be identified and mitigated against. The arbitrary threshold of 72 h used in this study is longer than the 48 h threshold used in other European/USA studies, but it is representative of clinical practice in Italy and helps contextualize the ongoing evolution of the perioperative management of colorectal patients. Indeed, in our study, the rate of early discharge (within 72 h) increased annually, with only a slight reduction in the early discharge rate in the first part of 2020, which may be attributed to new organizational issues that arose during the COVID-19 pandemic, increasing the difficulty for readmission due to the need for repeated SARS-CoV-2 testing and the presence of specific emergency room pathways for patients with fever^22^. The Italian healthcare system may also have hindered the shift towards early discharge (72 h) owing to the diagnostic related group (DRG) system regulations related to reimbursement for different procedures managed on a regional basis^23,24^. Considering all these issues, the modifiable factors that could be evaluated regarding the anticipation of discharge are surgeons’ perceptions and patients’ expectations in terms of comfort at the time of discharge, as well as fear of encountering management issues if an outpatient complication occurs.

The main limitation of this study is its retrospective single-centre design. The data were prospectively collected, but we were unable record all possible variables needed to investigate the discordancy between RfD and the date of discharge extensively. This includes information on the home and family environment that might impact on the discharge decision. In the future, more extensive prospective data collection and/or a study with a prospective design would allow to better investigation of the topic.

In the context of the application of the ERAS principles, early discharge (within 72 h or less) was proven to be safe and feasible, and was not associated with a high risk of readmission or reoperation in the study population. There is a subset of patients in whom discharge could be anticipated to be within 48 h with no significant risk for severe complications or readmission. Surgeons’ bias seems to affect the indication for discharge and is linked to a perceived high risk of comorbidities, the complexity of surgery, and possible management issues, even though these factors have minimal impact on the clinical outcomes. Future studies should prospectively compare the 48 h and 72 h discharge periods in terms of safety, as well as patients’ expectations and satisfaction, possible organizational issues, and means to reduce costs effectively in Italian clinical practice.

*Disclosure*. The authors declare no conflict of interest.

## Supplementary Material

zrac002_Supplementary_Data
