# Supplementary material for: Feasibility of discharge within 72 hours of major colorectal surgery: lessons learned after 5 years of institutional experience with the ERAS protocol
Source: BJS Open. 2022 Feb 16;6(1):zrac002. doi: 10.1093/bjsopen/zrac002 (PMC8855525; doi:10.1093/bjsopen/zrac002)
Supplement: zrac002_Supplementary_Data [file zrac002_supplementary_data.zip › zrac002_Supplementary_Data/Supplementary_Table_1.docx]

| Table S1. Short-term outcomes of patients in the DD and DND groups after discharge | | | |
| --- | --- | --- | --- |
| Variable | DD | DND | p-value |
| n = | 284 (36.6%) | 491 (63.4%) |  |
| Readmission n, (%) *  No  Yes | 274 (96.8)  9 (3.2) | 470 (95.9)  20 (4.1) | 0.525 |
| Reintervention n, (%)  No  Yes | 278 (97.9)  6 (2.1) | 482 (98.2)  9 (1.8) | 0.785 |
| Mortality after discharge**  No  Yes | 282 (99.3)  2 (0.7) | 491 (100)  0 (0) | 0.134 |

* 1 patient in the DD and 1 patient in the DND group were not able to recall if they were readmitted within 30 days from discharge; **3 patients died without being discharged were excluded from this analysis; % expressed by column.
