# Supplementary material for: Feasibility of discharge within 72 hours of major colorectal surgery: lessons learned after 5 years of institutional experience with the ERAS protocol
Source: BJS Open. 2022 Feb 16;6(1):zrac002. doi: 10.1093/bjsopen/zrac002 (PMC8855525; doi:10.1093/bjsopen/zrac002)
Supplement: zrac002_Supplementary_Data [file zrac002_supplementary_data.zip › zrac002_Supplementary_Data/Supplementary_Table_2.docx]

| Table S2. Multivariable logistic regression for factors associated with the DND status in patients without postoperative complications | | | | | |
| --- | --- | --- | --- | --- | --- |
| Variable | Beta | p-value | OR | CI 95% Low | CI 95% High |
| CCI | 0.122 | 0.024 | 1.130 | 1.016 | 1.257 |
| Mini-invasive surgery | 0.644 | 0.020 | 1.904 | 1.105 | 3.278 |
| Right hemicolectomy | -0.506 | 0.022 | 0.603 | 0.391 | 0.930 |
| Rectal resection | -0.499 | 0.065 | 0.607 | 0.357 | 1.032 |
| Diverting ileostomy | 0.543 | 0.094 | 1.722 | 0.912 | 3.249 |

Variables: Residence outside Rome, Residence outside Lazio, Age, Gender, BMI, CCI, ASA score, Mini-invasive surgery, Type of surgery, Stoma creation, duration of surgery, ICU postoperative stay, drain positioning, OR: odds ratio, CI: confidence interval.
